# Utilization of Micro-Doppler Radar to Classify Gait Patterns of Young and Elderly Adults: An Approach Using a Long Short-Term Memory Network

**DOI:** 10.3390/s21113643

**Published:** 2021-05-24

**Authors:** Sora Hayashi, Kenshi Saho, Keitaro Shioiri, Masahiro Fujimoto, Masao Masugi

**Affiliations:** 1 Graduate School of Science and Engineering, Ritsumeikan University, Shiga 525-8577, Japan; ri0063ir@ed.ritsumei.ac.jp (S.H.); masug@fc.ritsumei.ac.jp (M.M.); 2 Graduate School of Engineering, Toyama Prefectural University, Toyama 939-0398, Japan; t954005@st.pu-toyama.ac.jp; 3 Human Augmentation Research Center, National Institute of Advanced Industrial Science and Technology, Chiba 277-0882, Japan; masahiro-fujimoto@aist.go.jp

**Keywords:** Doppler radar, gait classification, machine learning, LSTM

## Abstract

To develop a daily monitoring system for early detection of fall risk of elderly people during walking, this study presents a highly accurate micro-Doppler radar (MDR)-based gait classification method for the young and elderly adults. Our method utilizes a time-series of velocity corresponding to leg motion during walking extracted from the MDR spectrogram (time-velocity distribution) in an experimental study involving 300 participants. The extracted time-series was inputted to a long short-term memory recurrent neural network to classify the gaits of young and elderly participant groups. We achieved a classification accuracy of 94.9%, which is significantly higher than that of a previously presented velocity-parameter-based classification method.

## 1. Introduction

The ability of elderly people to maintain their balance during walking is related to the risk of future falls and dementia [1,2]. Biomechanical studies have verified significant gait differences between participant groups with different fall risks, such as young adults, elderly adults, and elderly adults with a history of falls [3,4]. The use of daily gait measurements to detect age-related gait changes is an effective way for the early detection of the risks of dementia and falling accidents. Many techniques have been developed and widely used to conduct gait analysis of walking people. These techniques use optical motion capture, acceleration sensors, and a combination of a video camera and markers [3,5]. However, they require markers or devices to be attached to the subjects. Consequently, they are not appropriate for daily measurements. Some researchers studied the suitability of optical camera- and depth sensor-based techniques for daily measurements [6]. However, the accuracy of such methods depends on the lighting and clothing conditions.

Radar-based gait measurement methods were proposed to address the drawbacks of other sensor-based techniques [7,8,9,10,11,12,13,14,15,16,17,18]. Radar-based methods can remotely measure the velocity of whole human body parts without placing any constraints on the participant. In addition, they are unaffected by lighting or clothing conditions. Recently, micro-Doppler radar (MDR) has achieved accurate motion recognition and classification for various problems based on the deep-learning approach [7,8]. Because the MDR can measure micro-motions of humans, it is used for the recognition of detailed motions such as vital signs [9], gesture classification [10], and classifications of human gait types (e.g., classification with/without arm swinging [11] and slow/fast walking [12]). In addition, MDR-based techniques have been applied to obtain detailed gait measurement data that are used for personal identification [13,14] and the identification of gait type for rehabilitation and hospital applications [15,16]. However, the above-mentioned conventional studies have not focused on gait classification based on age-related gait changes, which are investigated in the field of biomechanics. Therefore, to apply the findings in biomechanics to gait classification using the MDR technique, we propose a method that uses the velocity parameters extracted from MDR measurement data obtained during walking in a previous work to classify two subject groups (young and elderly adults) based on their gait differences [17,18]. The support vector machine (SVM)-based results confirmed that our method could classify the two subject groups with high accuracy using the extracted velocity parameters. However, our previous method only utilized the velocity parameters, which do not provide comprehensive information regarding the time-series velocity fluctuations existing in the radar measurement data.

The proposed MDR measurement-based method considers the movement of body parts during walking, reflecting balance ability and the classification of young and elderly adult gait patterns. First, we extracted the time-series data that reveal how the velocities of the body and legs vary with the measured radar signals, with reference to the previously reported findings [17,18,19]. Subsequently, the extracted time-series data were inputted to a long short-term memory (LSTM) recurrent neural network [20]. Here, the input data were a combination of all three types of respective velocity time-series data. These velocity time-series data corresponded to the velocity fluctuations of the stepping leg during the stance phase, torso, and axis leg during the stance phase, respectively. The classification accuracies of young and elderly people groups were compared using each velocity time-series data. The results confirmed that our proposed method yields a classification accuracy of 94.9%, which is significantly higher than that of our previous method. Furthermore, our results significantly exceeded the accuracy achieved using combination technologies that use a video camera and markers presented in [5], which reported a maximum classification accuracy of 90%. To the best of our knowledge, this is the first study to yield a classification accuracy of over 90% for the radar-based gait classification of young and elderly adults.

## 2. Experimental Work

The study participants were 87 young (mean age: 22.0 ± 1.7 years, mean height: 169.7 ± 8.7 cm) and 213 elderly (mean age: 72.5 ± 4.8 years, mean height: 156.3 ± 8.6 cm) adults. None of the participants had any history of neurological, musculoskeletal, or other medical conditions, and they could safely walk without assistance. The elderly adults that participated in this study had a Mini-Mental State Examination score [21] of at least 24 points and a Timed Up and Go test [22] score of 13.5 s or less.

**Figure 1:**
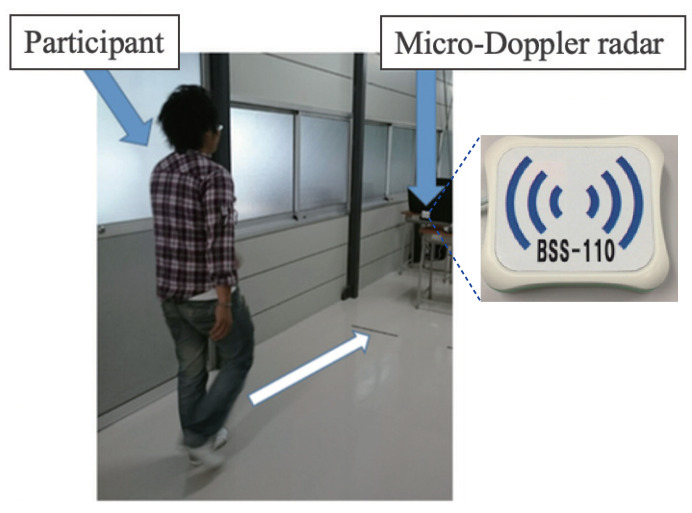
MDR experimental site for gait measurements.

Figure 1 shows our MDR experimental setup. The contentious-wave MDR (ILT Office BSS-110) operated at a frequency of 24 GHz was used. The participants walked toward the MDR along a 10 m walkway at self-selected comfortable speeds. No restrictions were imposed on the subjects’ clothes. The fixed mono-static MDR was installed in front of the participants at a height of 0.86 m. The radar specifications were as follows:

- Waveform of transmitting wave: Sinusoidal (24 GHz).
- Equivalent isotropically radiated power: 40 mW.
−3 dB beamwidth in E-plane: 70°.
−3 dB beamwidth in H-plane: 28°.
- Detection scheme of received signals: Coherent detection.
- Sampling frequency of received signals: 600 Hz.

**Figure 2:**
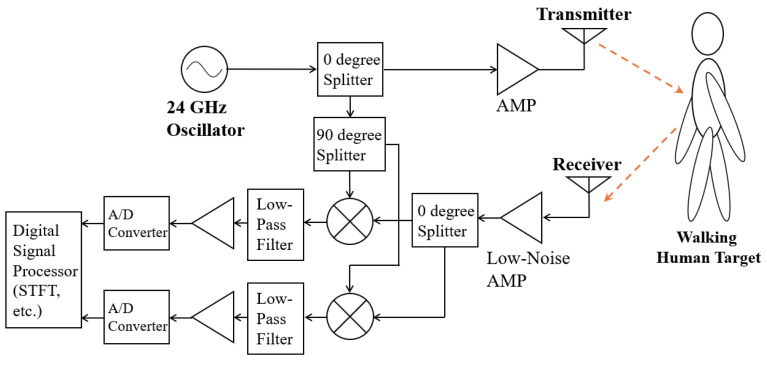
Block diagram of the MDR system.

The time-velocity distributions (spectrograms) of the MDR received signals were calculated. First, the received signals were obtained by the MDR system shown in Figure 2. The MDR transmitted sinusoidal waves at a frequency of 24.0 GHz onto a pedestrian participant. Then, the received signals after demodulation of the reflected waves were obtained.

The details of the acquisition of the spectrogram are as follows [23]. First, the transmitting signal of MDR is expressed as

(1) $$s_{T}\left(t\right)=Ae^{j(2\pi f_{0}t+\phi_{0})}$$

where *A* is the amplitude, $f_{0}$ is the frequency, and $\phi_{0}$ is the initial phase. The reflected echo from a point scatterer has a time-varying phase and can be expressed as [23]

(2) $$s_{R}^{\prime }\left(t\right)=\eta Ae^{j[2\pi f_{0}t+\phi_{0}+\phi \left(t\right)]},$$

where η < 1 is the ratio of the received level to *A*. Consequently, the reflected signal after demodulation from *N* multiple scatters is expressed as [7,23]

(3) $$s_{R}\left(t\right)=\sum_{i=1}^{N}\eta_{i}Ae^{j\phi_{i}\left(t\right)}.$$

The distance between the radar and the *i*-th scatterer is defined as $R_{i}$, and the radial velocity of the scatterer is defined as $v_{di}$. With these parameters, $\phi_{i}\left(t\right)$ is expressed as

(4) $$\phi_{i}\left(t\right)=-2\pi \left(\frac{2R_{i}}{\lambda }-\frac{2v_{di}t}{\lambda }\right),$$

where λ = $c/f_{0}$ is the wavelength and *c* is the speed of light. The Fourier transform of $f_{R}\left(t\right)$ is expressed as [23]

(5) $$s_{R}\left(f\right)=\sum_{i=1}^{N}\eta_{i}A\delta \left(f-f\mathrm{di}\right),$$

where η(f) is Dirac delta function and $f_{d}$ is called the Doppler frequency, which is expressed as follows:

(6) $$f_{\mathrm{di}}=\frac{2v_{\mathrm{di}}}{\lambda }$$

Based on (5) and (6), the short-time Fourier transform (STFT) of $s_{R}\left(t\right)$ derives its time-velocity distribution as [7]

(7) $$S_{R}\left(t,v_{d}\right)=\int s_{R}\left(t+\tau \right)W\left(\tau \right)d\tau ,$$

where W(t) is the window function. Because the spectrograms $|S_{R}\left(t,v_{d}\right){|}^{2}$ represent comprehensive gait characteristics corresponding to body parts, we extracted features to classify gaits using $S_{R}\left(t,v_{d}\right)$. For the STFT, we empirically used the Hamming window function with the length of 128 samples for W(t) of (7). In this study, two walking cycles of a participant’s gait in the steady state were set as an analysis interval for STFT. Therefore, the data length varies with participants, and is in the range of 99–224 data points.

## 3. Gait Classification Method

This section presents our gait classification method based on the velocity time-series extracted from the spectrograms obtained from the MDR gait measurements. The procedure of our gait classification method is as follows.

**Figure 3:**
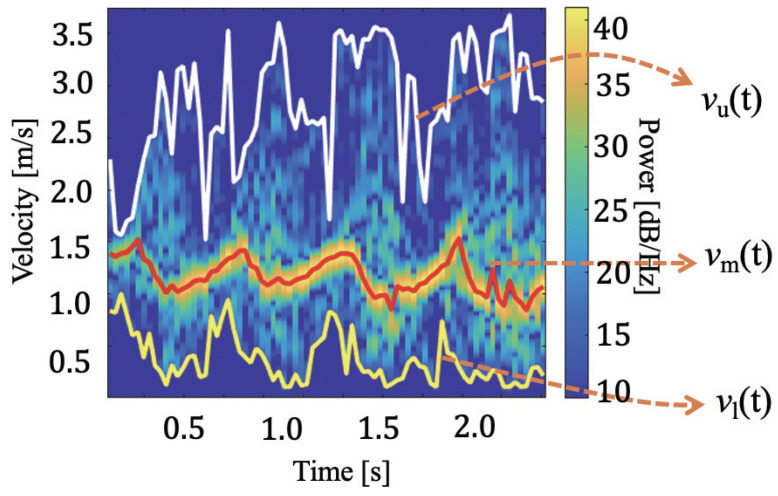
Example of the MDR spectrogram and extraction of envelopes.

**Figure 4:**
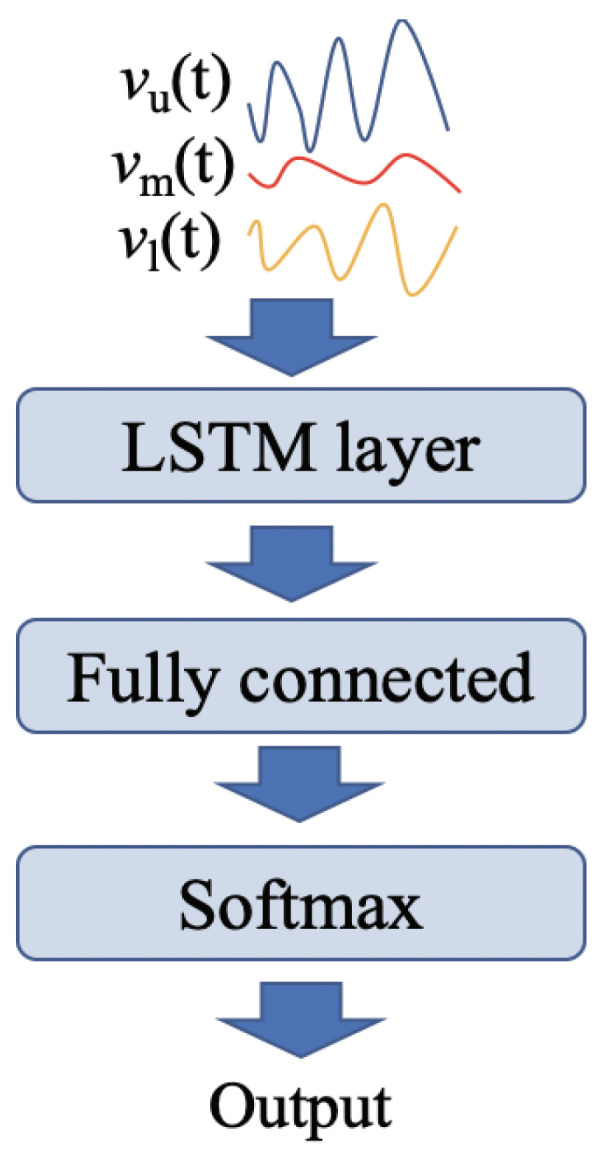
Outline of the proposed method. The extracted envelope(s) shown in Table 1 is inputted to the LSTM layer, and the general structure of the LSTM is used. The output is the classification result (Young or Elderly labels).

1. Calculation of STFT of the received signal (Figure 3 shows an example).
2. Extraction of feature envelopes from the STFT spectrogram ($v_{u}\left(t\right)$, $v_{m}\left(t\right)$, and $v_{l}\left(t\right)$ in Figure 3 ).
3. Inputting the feature envelopes to the LSTM network (Figure 4).

Figure 3 presents an example of a gait spectrogram and extraction of the feature envelopes. The upper envelope $v_{u}\left(t\right)$ corresponding to the maximum velocity, the lower envelope $v_{l}\left(t\right)$ corresponding to the minimum velocity, and the power-weighted mean velocity (mean envelope) $v_{m}\left(t\right)$ are extracted using a similar technique as that used in previous studies [17,18,19]. First, the mean envelope is obtained with the power-weighted mean velocity for each time *t*, expressed as [24]

(8) $$v_{m}\left(t\right)=\frac{\int v_{d}{\left|S_{R}\left(t,v_{d}\right)\right|}^{2}dv_{d}}{\int |S_{R}\left(t,v_{d}\right){|}^{2}dv_{d}}.$$

Then, significant peaks of the spectrogram are extracted. The significant peaks at time *t* are defined as peaks with relatively large received power, and they satisfy the following conditions [25]:

(9) $$\frac{d|S_{R}\left(t,v_{d}\right)|}{dv_{d}}=0,$$

(10) $$|S_{R}\left(t,v_{d}\right)\left|>\rho \max\right|S_{R}\left(t,v_{d}\right)|,$$

where 1>ρ>0 is the ratio of the peak extraction threshold amplitude to the maximum amplitude at each time *t*. We empirically set ρ=0.2. The extracted *m*-th significant peak velocity at time *t* is defined as $v_{\mathrm{dp},m}\left(t\right)$. Then, the upper and lower envelopes are extracted as the maximum and minimum velocities, respectively, in $v_{\mathrm{dp},m}\left(t\right)$ [19]:

(11) $$v_{u}\left(t\right)=\max_{m}v_{\mathrm{dp},m}\left(t\right),$$

(12) $$v_{l}\left(t\right)=\min_{m}v_{\mathrm{dp},m}\left(t\right).$$

The upper envelope, lower envelope, and power-weighted mean velocity mainly correspond to the swing phase, stance phase, and torso velocity, respectively.

In our proposed method, the velocity time-series of $v_{u}\left(t\right)$, $v_{l}\left(t\right)$, and $v_{m}\left(t\right)$ were inputted to the LSTM network, and the classification label (young or elderly) was output by the LSTM network. We consider combinations of these velocity time-series data inputted to the LSTM (Table 1), to clarify the efficient input time series for the gait classification. Note that, in our previous study [17], we derived simple parameters (e.g., mean and standard deviation) from the extracted time-series, and the obtained parameters were used for SVM-based discriminant analysis. Rather than using several parameters, our proposed method in this study uses time-series data as the input for the LSTM network.

The implantation of the LSTM is presented here. We employed a sequence-to-label classification scheme that uses one-label information with respect to time-series data for machine learning. Figure 4 illustrates the outline of the proposed classification method using the LSTM. The LSTM structure includes a sequence input layer, an LSTM layer, a fully connected layer, and a Softmax layer. In the sequence input layer, three types of velocity time-series data were inputted to the LSTM as one dataset. As mentioned previously, the data length inputted to the LSTM varies with participants. During the training process, the software performed zero-padding so that datasets have the same length (corresponding to the maximum length) for each divided batch. The LSTM layer learns the long-term dependencies between the time steps of the time-series data from the input dataset [26]. In addition, we employed the Adam optimization function. The hyper-parameters of the number of hidden cells, batch size, and initial learning rate were set to 400, 128, and 0.0001, respectively. The learning rate was attenuated every 30 epochs, with an attenuation multiplier of 0.9. These hyper-parameters were tuned empirically.

## 4. Result Evaluation and Discussion

### 4.1. Accuracy Evaluation and a Comparison between All Input Conditions

We employed the hold-out validation, which randomly divides the input dataset into training and testing datasets, to evaluate the results. Three velocity time-series datasets extracted from the spectrogram ($v_{u}\left(t\right)$, $v_{m}\left(t\right)$, and $v_{l}\left(t\right)$) of all the young and elderly people were randomly divided to realize a training-to-test data ratio of 7:3 (i.e., the number of training and test data were 210 and 90, respectively). After training the LSTM model, the test data were applied to the trained LSTM model to evaluate the classification accuracy. The average classification accuracy was calculated after performing 30 trials on the test data. We compared the classification accuracies using the input conditions of Table 1.

Table 2 shows the results of the classification accuracy corresponding to the combination for each velocity time-series data. Each classification accuracy represents the average of 30 training trials. As shown in this table, Condition 3 that inputs only $v_{u}\left(t\right)$ exhibited the highest classification accuracy of 94.9%; Conditions 6 and 7 that consider the combination of extracted velocity time series also exhibited accuracies of over 90%. Table 3 shows the confusion matrices for Conditions 3, 4, and 7 calculated using 30 test trials. Under all conditions, the error rates corresponding to the misclassified elderly participants to that of young participants were relatively large. However, correct classifications with accuracies over 80% were confirmed for both classes in all conditions. In particular, Condition 3 achieved an accuracy of over 90% for both classes.

The above classification results indicate that age-related gait changes can be accurately detected based on the velocity time series of the legs’ motion extracted from the MDR. In biomechanics studies, age-related gait changes are important to evaluate fall risks during walking because aging is associated with gait slowing [27,28]. Thus, our method can be applied to monitoring systems for the early detection of individuals with high risks of falling.

Furthermore, our results demonstrate that the velocity time series extracted as the lower envelope of the spectrogram is more effective than those extracted as the upper and mean envelopes. Similar to our previous study [17], the upper and mean envelopes also can classify the young and elderly adults with sufficiently high accuracies of over 80%. We now discuss the reasons for these results. The young and elderly adults were classified based on the differences in their legs’ motions that are reflected in the gait acceleration, which were discussed in our previous studies [3,17]. Ref. [3] reported that a significant difference between young and elderly adults in gait was detected in the acceleration compared with the gait speed. Based on this, we can predict that $v_{m}\left(t\right)$ is relatively inefficient for their classification because $v_{m}\left(t\right)$ corresponds to body motion and reflects gait speed. In contrast, $v_{u}\left(t\right)$ and $v_{l}\left(t\right)$ include information to achieve classification accuracy because the gait acceleration is closely related to the legs’ motions [17]. Thus, the classification accuracy obtained using only $v_{m}\left(t\right)$ (Condition 2) is relatively lower than that using $v_{u}\left(t\right)$ and/or $v_{l}\left(t\right)$ (other conditions). However, this study revealed the highest effectiveness of the lower envelope when we used the information of the entire time series of envelopes for the LSTM-based classification. This may be because the significant differences in the gait parameters in the stance phase between the young and elderly adults [28] were efficiently extracted in the lower envelope. However, further studies are required to clarify its mechanism.

### 4.2. Comparison with the Previous Method Results

**Figure 5:**
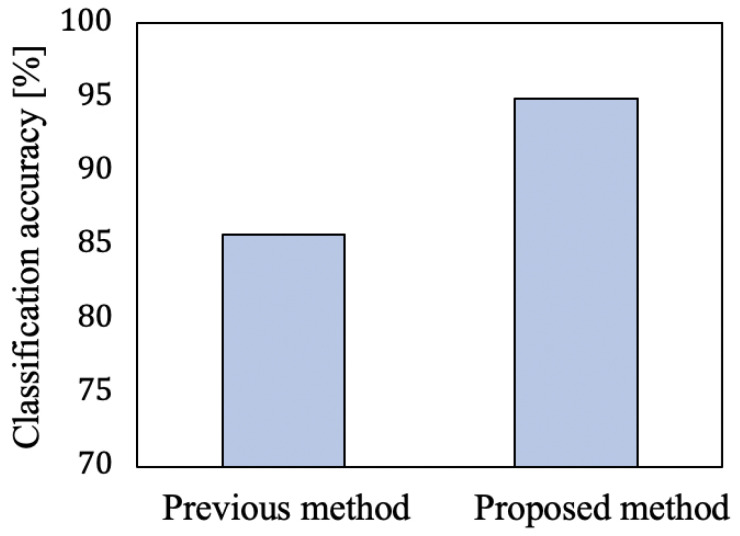
Classification accuracies using the conventional and the proposed methods.

First, we evaluated the accuracy of the previous method presented in [17] (SVM-based classification using the velocity parameters) in the same manner to compare with the proposed method. The proposed method used Condition 3, which inputted only the lower envelope $v_{l}\left(t\right)$ to the LSTM and achieved superior accuracy, as shown in Table 2. Figure 5 summarizes a comparison of the results of the previous and proposed methods. The average classification accuracies, achieved by the proposed and previous methods, were 94.9% (for Condition 3) and 85.7%, respectively. These results confirmed that the classification accuracy of the proposed method was significantly higher than that of the previous method.

The previous method only uses the representative parameters of the extracted envelopes, such as mean and standard deviation. Thus, this approach could not sufficiently extract the essential information on the gait differences in the young and elderly adults. In contrast, the proposed method efficiently uses the comprehensive information of the time series using the LSTM and, thus, achieved highly accurate classification.

### 4.3. Application of the Convolutional Neural Network-Based Method

Finally, we investigated the application of convolutional neural network (CNN)-based classification method [7,14], which is the state-of-the-art method of motion classification using MDR data, to the gait classification of young and elderly adults. In this method, the spectrograms are converted to images, and these images are inputted to the CNN for classifying the participants. We used the AlexNet architecture [29] for the CNN, which is one of the representative networks of CNN, and its hyper-parameters were empirically optimized similar to the LSTM. Additionally, we investigated the efficiency of areas in the input spectrogram images using a gradient-weighted class activation mapping (Grad-CAM) visualization [30]. Grad-CAM visualizes critical regions in input images by producing a heat map showing the importance of classification. Similar to the LSTM, the classification accuracy was evaluated using 30 trials of hold-out validations with a training-to-test data ratio of 7:3.

**Figure 6:**
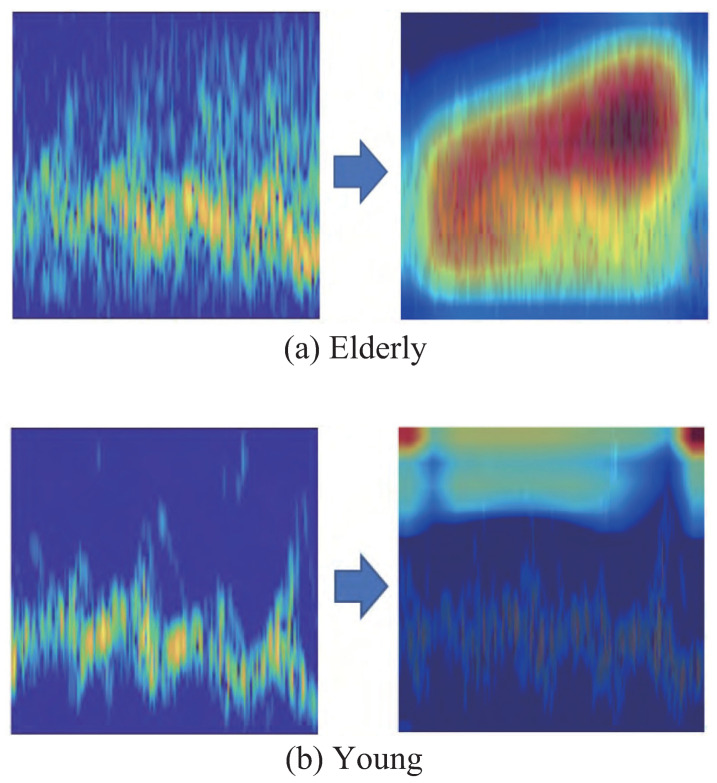
Grad-CAM visualization results for the elderly (**a**) and young (**b**) adults. **Left**: original input spectrogram image; **Right**: Grad-CAM heat map (red color indicates a high score of importance in the classification).

The classification accuracy was 97.8%, which is higher than that of the proposed LSTM-based method. However, in assessing the results of the Grad-CAM visualization, this high accuracy is not necessarily reliable (examples of obtained heat maps are shown in Figure 6). Although the Grad-CAM visualization for elderly adults indicated that the components corresponding to gait movement were efficiently used, that for the young adults indicated that the background components were judged as the important area. In our data collection, the MDR experiments for the young and elderly adults were performed in different places, and their characteristics of measurement noises varied. The elderly data included a relatively large number of components corresponding to noise. Thus, the CNN positively used this difference of background noises for classification. Note that the Grad-CAM visualization results of many other participants indicated a comparable tendency.

In contrast to the CNN, the LSTM-based approach was relatively unaffected by such background noises because the components corresponding to the background noises were cancelled in the peak extraction process for the extractions of envelopes ((11), (12), and (10)). Therefore, the results of the LSTM used the envelopes (time series of velocities) corresponding to the main components of walking motion and our method efficiently classified the gaits.

## 5. Conclusions

In this study, we presented an LSTM-based method using the MDR signals for the classification of young and elderly adult gaits. The velocity time-series data reflecting each body part (the stepping leg in the stance phase, torso, and leg in the stance phase) were extracted as the envelopes of the MDR spectrograms and inputted to the LSTM for classification. The experimental results confirmed a classification accuracy of 94.9% with the use of velocity time-series corresponding to the motions of legs contacted to the ground in the stance phase. This study is the first to achieve a classification accuracy of over 90% for a radar-based method for the gait classification of young and elderly adults. The achieved accuracy is approximately 9% higher than that of our previous method, which utilized the velocity parameters extracted from the spectrograms and SVM.

In the future, we plan to analyze the gait characteristics of participants, including a middle-aged participant group, and evaluate the network performance with a larger dataset. Furthermore, the application of the fuzzy time-series approach [31] to our proposed method can be considered as efficient because the extracted envelopes include the uncertainty and/or imprecision, which can be efficiently dealt with using fuzzy modeling. In particular, recently proposed fuzzy time-series models [32,33] could possibly improve the robustness and accuracy of the proposed method; therefore, investigating their applicability is an important direction of future study.

## Figures and Tables

**Table 1:** Combinations of velocity time-series data inputted to the LSTM.

Condition	Input Velocity Time-Series Data for CNN
Condition 1	$v_{u}\left(t\right)$
Condition 2	$v_{m}\left(t\right)$
Condition 3	$v_{l}\left(t\right)$
Condition 4	$v_{u}\left(t\right)$, $v_{m}\left(t\right)$
Condition 5	$v_{u}\left(t\right)$, $v_{l}\left(t\right)$
Condition 6	$v_{m}\left(t\right)$, $v_{l}\left(t\right)$
Condition 7	$v_{u}\left(t\right)$, $v_{m}\left(t\right)$, $v_{l}\left(t\right)$

**Table 2:** Classification results for all combinations of velocity time-series data.

Condition	Classification Accuracy
Condition 1	84.9%
Condition 2	83.3%
Condition 3	94.9%
Condition 4	87.0%
Condition 5	88.6%
Condition 6	92.0%
Condition 7	92.7%

**Table 3:** Confusion matrices for tests in Conditions 3, 4, and 7. x/y/z in each element denotes the results of Condition 3/4/7 (%).

Predicted Class\True Class	Young	Elderly
Young	91.7/84.4/85.1	8.3/15.6/14.9
Elderly	2.2/11.4/4.8	97.8/88.6/95.2

## Data Availability

The data presented in this study are available on request from the corresponding author. The data are not publicly available due to privacy issues.

## Abbreviations

The following abbreviations are used in this manuscript:

MDR	Micro-Doppler radar
SVM	Surpport vector machine
LSTM	Long short-term memory
